# Sexual Stimulation Increases the Survival of New Cells in the Accessory Olfactory Bulb of the Male Rat

**DOI:** 10.3389/fnins.2016.00065

**Published:** 2016-03-01

**Authors:** Nancy M. Unda, Wendy Portillo, Rebeca Corona, Raúl G. Paredes

**Affiliations:** Neurobiología Conductual y Cognitiva, Instituto de Neurobiología, Universidad Nacional Autónoma de MéxicoJuriquilla, Mexico

**Keywords:** neurogenesis, sexual behavior, olfactory bulb, neuronal plasticity, male rats

## Abstract

Sexual behavior in rodents is modulated by the olfactory system. The olfactory bulb (OB) is a structure that undergoes continues neurogenesis in adulthood. We have previously shown that 15 days after males rats pace the sexual interaction and ejaculate 1 or 3 times, there is an increase in the density of new cells that reach the accessory olfactory bulb (AOB). The aim of the present study was to evaluate if sexual behavior in male rats increases the density of new neurons that survive 45 days after sexual behavior in the AOB and in the main OB (MOB). Male rats were randomly divided in four groups: (1) Control (Ctr), males without sexual interaction; (2) Exposed (Exp), males only exposed to a sexually receptive female; (3) No pacing (NP), males that mated in conditions in which the female paced the sexual interaction; (4) One ejaculation (1E), males that paced the sexual interaction with a receptive female and ejaculated once; and (5) Three ejaculations (3E), males that paced the sexual interaction and were allowed to ejaculate three times. All males were injected with the DNA synthesis marker 5-bromo-2-deoxyuridine (BrdU), and were tested in one of the above conditions. 45 days later they were sacrificed, and the OBs were processed to identify new cells and evaluate if they had differentiated into neurons. Our data indicate that males that ejaculated three times showed an increase in the density of new cells that survive in the posterior part of the granular cell layer of the AOB and have more new neurons that the control group. However, no significant differences were found in the percentage of new cells that differentiate into neurons. No significant increase in the density of new cells was observed in the MOB. Our data show that pacing the sexual interaction until three ejaculations increases the density of new cells and neurons in the granular layer of the AOB, confirming that sexual behavior induces long-lasting plastic changes in the OB.

## Introduction

In rodents, the olfactory system regulates neuroendocrine and reproductive functions, allowing animals to recognize conspecifics and to determine gender and hormonal condition (Tirindelli et al., 2009). Two systems, the main (MOS) and the accessory olfactory system (AOS) process odorants. The odorants are detected by the olfactory neurons that extend axons to the glomeruli and synapse onto dendrites of the mitral and periglomerular cells. The dendrites of periglomerular cells form reciprocal dendrodendritic synapses with dendrites of mitral cells. Granular interneurons modulate the activity of the mitral neurons, establishing microcircuits in the olfactory bulb (Murphy et al., 2005). Mitral cells of the main olfactory bulb (MOB) send their axons through the lateral olfactory tract to central regions in the brain such as the anterior olfactory nucleus, cortical and medial-anterior amygdala (AMG), olfactory tuberculus, piriform, and entorhinal cortex (Baum and Kelliher, 2009; Sosulski et al., 2011; Baum and Cherry, 2015).

The AOS is anatomically and functionally divided into anterior and posterior regions. Superficial vomeronasal neurons that express the vomeronasal receptor 1 (V1R) project axons to the anterior subdivision of the accessory olfactory bulb (aAOB). Deeper vomeronsal neurons that express the vomeronasal receptor 2 (V2R) project to the posterior subdivision of the AOB (pAOB; Herrada and Dulac, 1997; Rodriguez et al., 1999; Tirindelli et al., 2009). Mitral cells in the AOB project, in turn, to the vomeronasal AMG, which synapses onto the bed nucleus of the stria terminalis (BNST), medial preoptic area (MPOA), and ventro medial hypothalamus (VMH), among other brain centers (Kevetter and Winans, 1981; Baum and Kelliher, 2009). Functionally, exposure to volatile components of female urine activates the aAOB and to some extent the caudal part of the pAOB in the male rat, and stimulation with volatile components of male urine activates the aAOB in male and female rats. On the other hand, non-volatile components of female urine induce activation preponderantly in the pAOB and to a lesser extent in the aAOB (Sugai et al., 2006).

The MOS and AOS play crucial roles in the expression of male sexual behavior, since sexually experienced male rats with lesions of the vomeronasal organ showed alterations in sexual behavior. After the lesion, males took longer to intromit and ejaculate because they displayed more mounts but less intromission than sham lesioned animals (Saito and Moltz, 1986; Kondo et al., 2003). Male rats with lesions of the olfactory epithelium showed a decrease in the intromission frequency and an increase in the ejaculation and mount latencies (Dhungel et al., 2011).

It is well recognized that adult neurogenesis is a mechanism of adult brain plasticity. In rodents, the olfactory bulbs (OB) continuously add new cells that are generated in the subventricular zone (SVZ) and rostral migratory stream (RMS) (Stolp and Molnár, 2015). The new cells migrate tangentially along the RMS, and in 15–20 days they arrive at the OB (Petreanu and Alvarez-Buylla, 2002; Winner et al., 2002). Around 95% of the new cells differentiate into granular neurons and integrate in the granular cell layer (GrL), and a few differentiate into periglomerular neurons that are incorporated into the glomerular cell layer (GlL; Petreanu and Alvarez-Buylla, 2002; Lledo and Saghatelyan, 2005; Bagley et al., 2007; Whitman and Greer, 2009). Between 15 and 45 days after birth, the number of granular cells declines to half; in this period sensory stimulation is critical for their survival (Petreanu and Alvarez-Buylla, 2002; Winner et al., 2002), because the new cells mature morphologically and are incorporated into existing neural circuits (Petreanu and Alvarez-Buylla, 2002). Neurogenesis is a dynamic process that depends on internal and external environmental cues (Rochefort et al., 2002; Rochefort and Lledo, 2005; Mak et al., 2007; Larsen et al., 2008; Oboti et al., 2009). Recently, it was demonstrated that sexual stimulation can modulate neurogenesis in the dentate gyrus (DG) of the hippocampus. Male rats that mated once or several times showed increased cell proliferation and survival of the new cells in the DG of the hippocampus, compared to males exposed to non-receptive females or males without sexual experience (Leuner et al., 2010). Sakamoto and coworkers (Sakamoto et al., 2011) developed a transgenic mouse using tamoxifen-treated Nestin-CreER^T2^ neuron specific enolase-diphtheria toxin fragment A (NSE-DTA) that is not able to integrate new neurons. In this mouse, when the cells reach maturity the NSE promoter becomes active and induces the expression of DTA, killing the new neurons. Male mice with this mutation show a decreased frequency and duration of mounting and produce fewer vaginal plugs than control mice (Sakamoto et al., 2011).

New cells are also involved in mate recognition. Female mice form an olfactory memory of their mating partner; when a recently mated female is exposed to an unfamiliar male, a neuroendocrine reflex is triggered that leads to pregnancy block (Bruce effect). Female mice treated with the antimitotic drug cytosine arabinose (Ara-C) to abolish neurogenesis are not able to make a memory of their sexual partner, and cohabitation with the mate starts the pregnancy block (Oboti et al., 2011). In addition, administration of Ara-C to male rats decreases the intromission frequency and copulatory efficiency (total number of intromissions/sum of intromissions and mount frequency), and the males are not able to ejaculate (Lau et al., 2011). Thus, new cells play a relevant role in sexual behavior and mate recognition memory.

We previously demonstrated that male rats that pace the sexual interaction and ejaculate one or three times showed an increase in the density of new cells that reach the GrL of the AOB 15 days after sexual stimulation and some of these cells differentiate into neurons. Interestingly, no such increase was observed in those males that mated for 1 h and ejaculated around 3 times but were not allowed to pace the sexual interaction (Portillo et al., 2012). The aim of the present study was to evaluate if one sexual behavior session in male rats increases the density of new cells that survive in the OB for 45 days after mating, and if these new cells differentiate into neurons.

## Methods

### Subjects

Forty adult male *Wistar* rats (300–350 g) were used in this experiment. Sexually experienced females from the same strain were used as stimulus. The females were ovariectomized and hormonally primed with estradiol benzoate (25 μg/rat, 48 h, Sigma, St. Louis, MO, USA) and progesterone (1 mg/rat, Aldrich, St. Louis, MO, USA) both diluted in corn oil to induce sexual receptivity. All the animals had unlimited access to food and water and were maintained on a reverse 12-h:12-h light-dark cycle. Experiments were carried out in accordance with the “Reglamento de la Ley General de Salud en Materia de Investigacion para la Salud” of the Mexican Health Ministry that matches NIH guidelines for the use and care of animals and approved by the Instituto de Neurobiologia animal care committee.

### Behavioral test

Before the experiment, males were trained over 3 weeks to acquire sexual experience. They were tested once per week in 30-min sessions with a sexually receptive female. Those males that ejaculated once in each session were included in the study. All the behavioral tests were done in transparent acrylic cages (dimensions: 62 × 29 × 42 cm). The floor of the cages were covered with sawdust, which was changed between behavioral tests. After the screening tests, 40 sexually experienced males were obtained and randomly divided into five groups: (1) Control (Ctr), males that were placed in a clean cage; (2) Exposed (Exp), males only exposed to a sexually receptive female in a mating cage divided by an acrylic barrier with small holes. In this way the male could smell, see and hear the receptive female, but physical contact was not possible; (3) No pacing (NP), males that mated in conditions where the females paced the sexual interaction. Subjects were placed in a mating cage divided by a barrier with a small hole in the middle that allowed the female, but not the male, to go back and forth from the male compartment; (4) One ejaculation (1E), males that controlled the sexual interaction, mated in a mating cage without the barrier, and were allowed to ejaculate one time, and (5) Three ejaculations (3E), males that controlled the sexual interaction and were allowed to ejaculate three times. For all sexual behavior tests, females were placed in the cage 5 min before the males were introduced. The following parameters of sexual behavior were registered: number of mounts, intromissions and ejaculations; mount, intromission and ejaculation latencies, and the inter-intromission interval (ejaculation latency divided by the number of intromissions) and post-ejaculatory interval (latency of the first intromission after the ejaculation minus the ejaculation latency).

### BrdU administration

Male rats (*n* = 8 per group) were intraperitoneally injected with the DNA synthesis marker 5′-Bromo-2′-deoxyuridine (BrdU) (Sigma, dissolved in NaCl 0.9%) at three different times: the first dose was given 1 h before the behavioral test, the second dose at the end of the behavioral test and the third dose, 1 h after the end of the test. The dose of BrdU was 100 mg/Kg (total 300 mg/kg). This concentration of BrdU labels the maximal number of cells and is not toxic for the subject (Cameron and McKay, 2001). All experimental males were sacrificed 45 days after the behavioral test.

### Immunohistochemistry

Animals were anesthetized with an intraperitoneal injection of pentobarbital (63 mg/rat), and transcardially perfused with 200 ml 0.1 M phosphate buffer followed by 200 ml of 4% paraformaldehyde (PFA) in phosphate buffer. Brains were post-fixed 1 h in PFA and maintained in 30% sucrose (cryoprotector) until they were processed. The MOB and AOB were cut in sagittal sections of 30 μm thickness using a microtome (Leica).

To label the density of the new cells (BrdU positive), MOB and AOB slices were processed for immunohistochemistry following the procedure previously reported (Corona et al., 2011a; Portillo et al., 2012; Arzate et al., 2013). Briefly, brain slices were washed in a solution of Tris buffer (TBS) and incubated in 2N HCl for 1 h. To block non-specific sites, the tissue was incubated for 15 min in a solution containing 10% bovine albumin and 0.3% Triton X-100 (Tx), then washed in TBS and incubated with anti-BrdU mouse antibody (1:2000; BD Bioscience) for 16 h at 4°C. After the antibody, the tissue was washed in TBS, albumin bovine (1%) and Tx (0.02%) and it was incubated in biotinylated anti-mouse IgG antibody (1:500; Vector Laboratory Burlingame, CA) for 2 h. Later, the tissue was incubated for 90 min with the avidin-biotin complex (AB) followed by incubation with 0.02% diaminobenzidine (DAB). After rinsing, sections were mounted on slides, coverslipped using permount and left to dry before being analyzed under the light microscope.

### Immunofluorescence

In order to identify the neuronal phenotype of the new cells that reach the granular cell layer (GrL), double staining for BrdU, and the neuronal protein NeuN was used. Following the protocol previously described (Corona et al., 2011a; Portillo et al., 2012; Arzate et al., 2013), OB slices were incubated for 48 h at 4°C simultaneously with both primary antibodies rat anti-BrdU (1:800, AbD Serotec) and mouse anti-NeuN (1:250, MILLIPORE) and then, after washing, with secondary antibodies anti-rat IgG Alexa Fluor 488 (1:1250, Invitrogen) and anti-mouse IgG Alexa Fluor 568 (1:1250, Invitrogen), respectively. After rinsing, sections were mounted on slides and coverslipped using aqua poly/mount, a non-fluorescing aqueous mounting medium (Polysciences, Inc).

### Quantification of BrdU- and BrdU-NeuN positive cells

**Figure 1:**
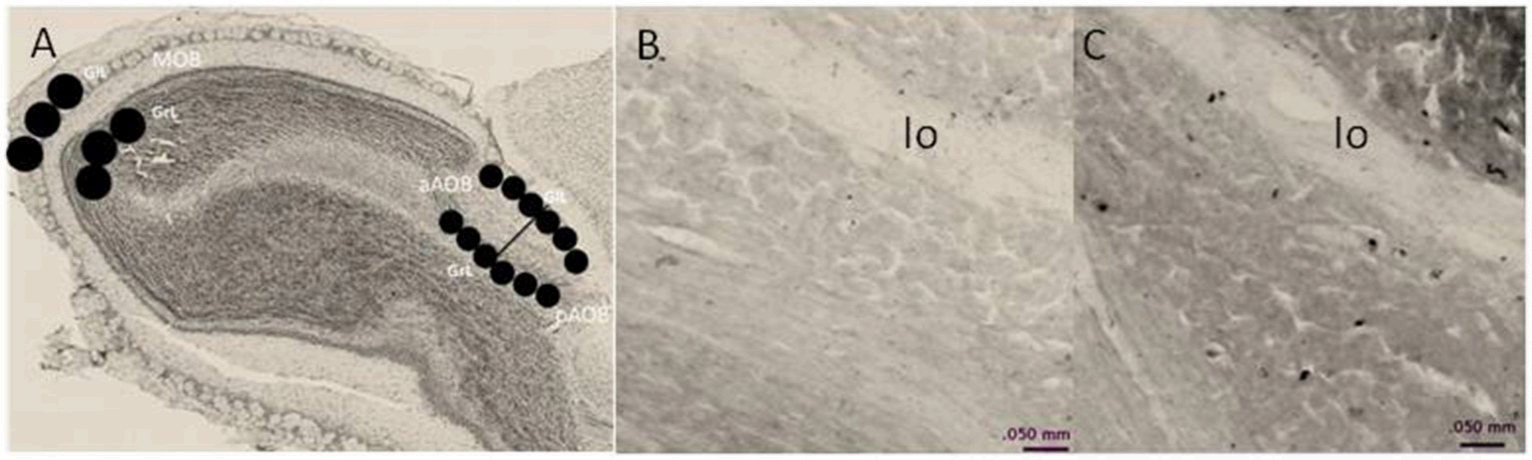
**(A)** Schematic representation of the regions in which BrdU-positive cells were counted. In the MOB, cells were counted in three, 400-μm diameter circles placed in the glomerular cell layer (GlL) and granular cell layer (GrL). The AOB was divided into the anterior (aAOB) and posterior (pAOB) regions, and cells were counted in the three 200-μm circles were placed in the GlL and GrL. **(B,C)** are representative photomicrographs (10X) of the BrdU-positive cells in the GrL of the pAOB in **(B)** control and **(C)** three ejaculation groups. lo lateral olfactory tract.

To quantify the density of BrdU-positive cells in the MOB and AOB, we used the software Image Pro Plus 6.1. Photomicrographs were taken with a light microscope OLYMPUS BX60 (10X objective). The BrdU-positive cells were quantified in the glomerular (GlL), and GrL of the MOB and AOB. To quantify the density of BrdU-positive cells in the MOB three circular areas of 400 μm diameter were placed in each layer. For the AOB, three circular areas of 200 μm diameter were placed in each layer of the anterior and posterior regions. We quantified four brain slices per animal. Circular areas for analysis are indicated in Figure 1.

To evaluate the percentage and density of BrdU-/NeuN-positive cells, images of 20X obtained with an inverted Zeiss LSM 780 confocal were analyzed. OB sections were sequentially scanned in a Z-stack analysis with a step of 0.8-μm thickness between each scan. The percentage and density of BrdU-/NeuN-positive cells was quantified in the GrL of the aAOB and pAOB for the Ctr and the 3E groups, because in this latter group we observed an increase in the density of BrdU-positive cells. Two AOB slices for each animal were analyzed.

### Statistical analysis

Data from the behavioral test were not normally distributed and therefore, they were analyzed by the Kruskal-Wallis test (K-W); in case of significant differences we used the Tukey Post hoc test. The density of BrdU-immunoreactive cells in the MOB, aAOB and pAOB was analyzed by a one-way analysis of variance (ANOVA), and the Tukey test was used as Post hoc test. The density of BrdU/NeuN positive cells was analyzed using a *t*-test and the percentage of BrdU/NeuN-immunoreactive cells were not normally distributed and therefore they were analyzed by a Mann-Whitney U test.

## Results

### Sexual behavior

Data from the sexual behavior test done when BrdU was administrated indicate that the males from the NP group show a higher inter-intromission interval than the 1E group [K-W: χ_(2)_2 = 9.96, *p* = 0.007]. No significant differences were found in the other parameters analyzed. Table 1.

**Table 1:** **Data from the sexual behavior test in the different groups: no pacing (NP), one ejaculation (1E), and three ejaculations (3E); *n* = 8 per group**.

**Behavioral parameters**	**NP**	**1E**	**3E**
No. Mounts	10.78±5.6	12.1±4.2	11.1±1.9
No. Intromissions	10.1±1.8	14.3±2.4	14.9±1.6
No. Ejaculations	2.6±0.4	1	3
Mount Lat.	177.3±65.3	174.1±32.4	120±32
Intromission Lat.	247±3	216.7±41.5	209.9±79.7
Ejaculation Lat.	769±164.4	634±150	754.8±108.8
PEI	457.3±45.3	413.4±21.5	383.8±16.6
III	75.2±7.5^*^	41.5±4	49.8±4

*Data were analyzed using the Kruskal-Wallis test; in case of significant differences the Tukey Post-hoc test was used. Data are expressed as the mean ± standard error. The latency (Lat) to the first mount, intromission and ejaculation, as well as the post-ejaculatory interval (PEI) and the inter-intromission interval (III) are expressed in seconds*.

* *Significantly different from 1E group. P < 0.05*.

### BrdU-positive cells

#### AOB

**Figure 2:**
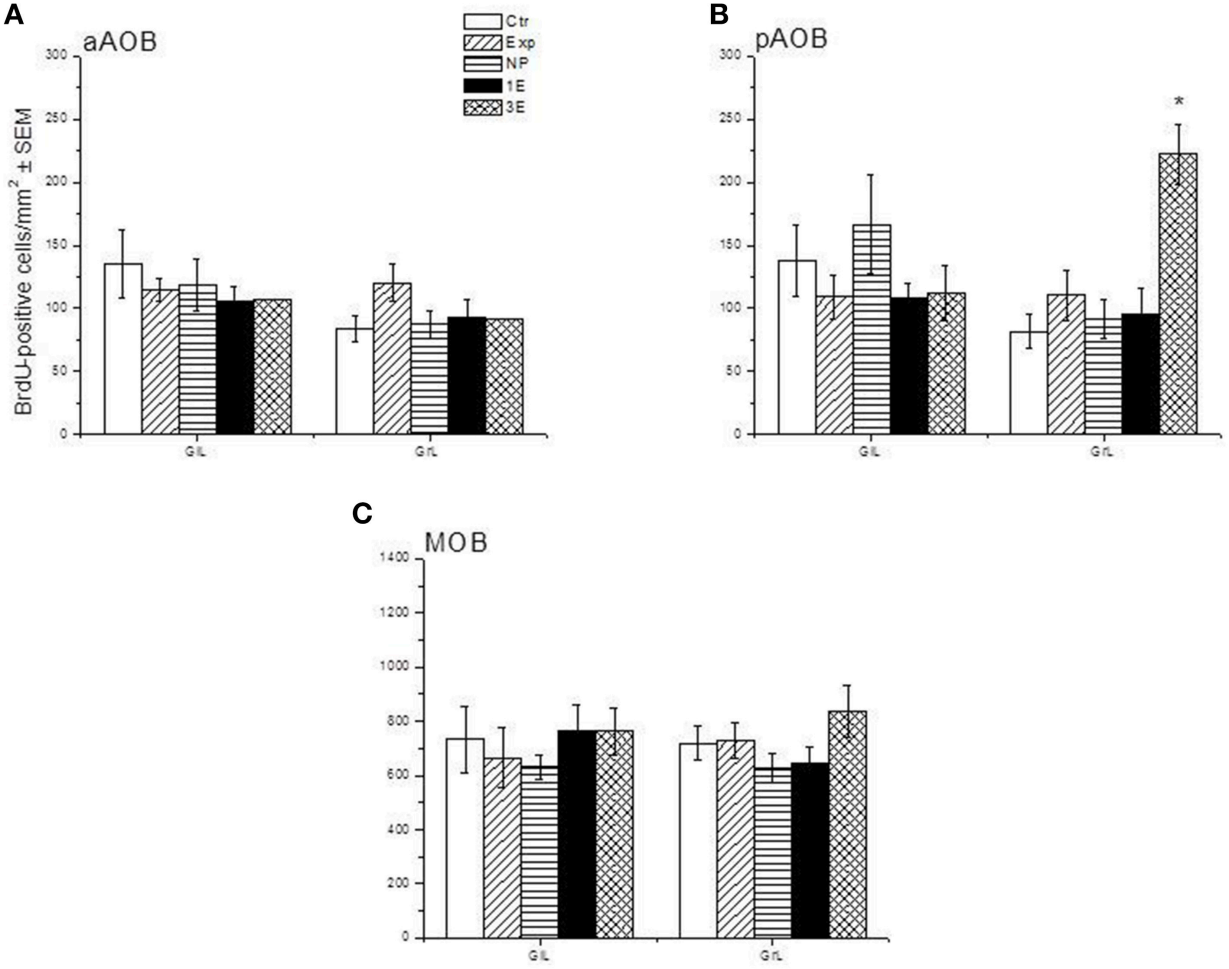
**Density of BrdU-positive cells in the sagittal plane in control (Ctr, *n* = 7), exposed (Exp, *n* = 8), non-paced (NP, *n* = 8), 1 ejaculation (1E, *n* = 6) and 3 ejaculation (3E, *n* = 8) groups**. The analyzed regions were the GlL and GrL of the aAOB **(A)**, pAOB **(B)**, and MOB **(C)**. Data are expressed as the mean ± SEM. ^*^Different from all other groups in the same layer. *P* < 0.05.

Representative photomicrographs of BrdU-positive cells in the pAOB are shown in Figure 1. Significant differences between the groups were found in the density of BrdU-positive cells in the GrL of the pAOB (*F* = 9.504, *p* = 0.001). The post hoc test revealed that the males from the 3E group had significantly more new cells that survive than the Ctr, Exp, NP and 1E groups. No significant differences were found in the GlL (*F* = 0.943, *p* = 0.452) of the pAOB, Figure 2B. Similarly, no significant differences were found in the GlL (*F* = 0.39, *p* = 0.809) and GrL (*F* = 1.302, *p* = 0.290) of the aAOB, Figure 2A.

#### MOB

No differences were observed in the density of cells that survive in the GlL (*F* = 0.427, *p* = 0.788) or GrL (*F* = 1.429, *p* = 0.247) of the MOB, Figure 2C.

### BrdU/NeuN-positive cells

**Figure 3:**
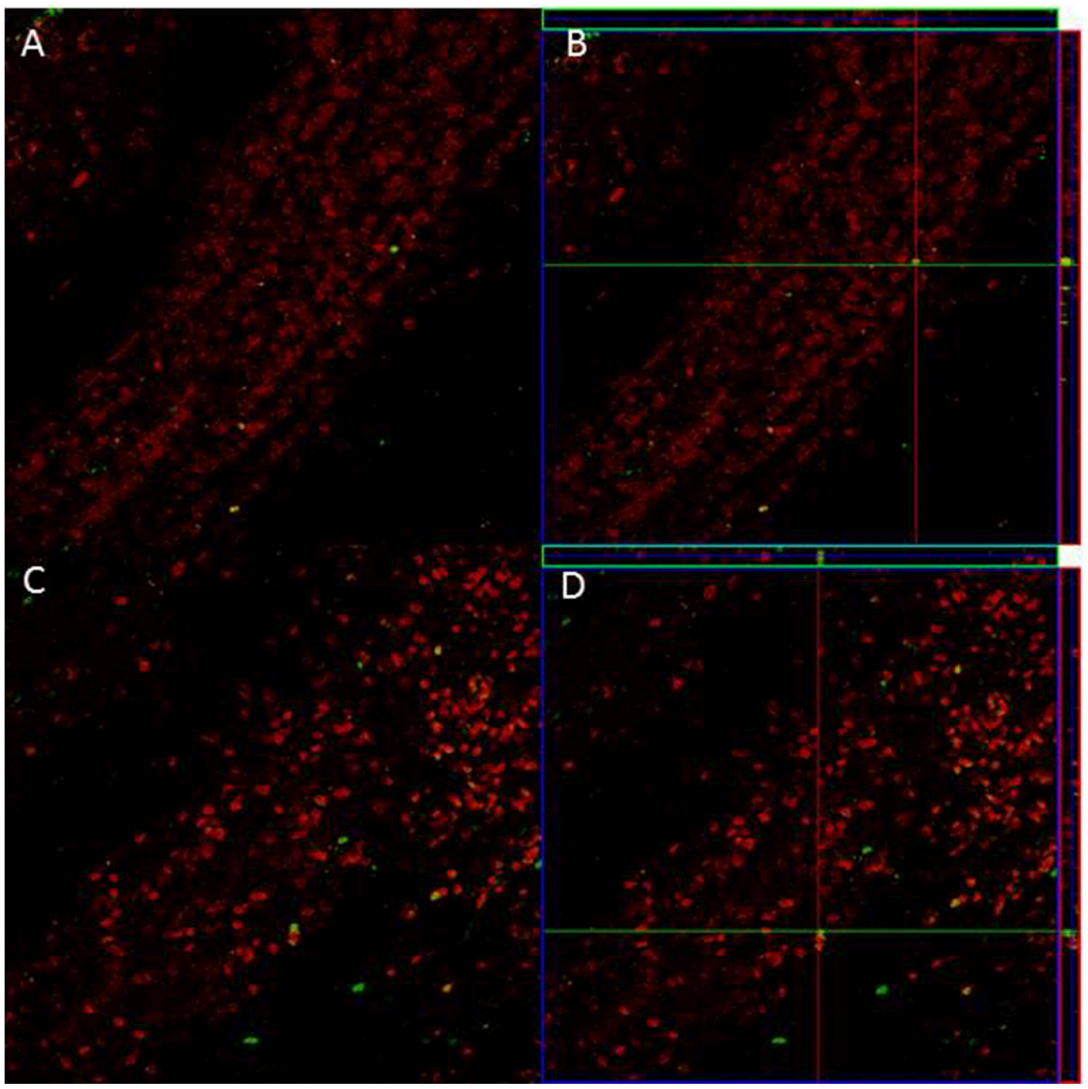
**Confocal images of cells in the GrL of the AOB, double-labeled with NeuN (red) and BrdU (green) in control (A) and 3E group (C)**. Projection and orthogonal plane in control **(B)** and 3E group **(D)**.

Since we only found significant differences in the density of new cells that survive in the GrL of the AOB, we evaluated if sexual behavior increased the density and percentage of new cells that differentiate into neurons in the 3E group in comparison to the control group. In the pAOB the 3E group had more BrdU/NeuN positive cells than the Ctr group (*t* = −2.8, *p* = 0.014). No significant differences were found in the density of BrdU/NeuN cells in the aAOB (*t* = −0.54, *p* = 0.6) Table 2. No differences were found in the percentage of new cells that differentiate into neurons in the anterior (*T* = 67, *p* = 0.96) and posterior (*T* = 54, *p* = 0.16) GrL of the AOB (Table 2). Representative photomicrographies are shown in the Figure 3.

**Table 2:** **Density and percentage of BrdU/NeuN positive cells in the GrL of the AOB in control (Ctr) and three ejaculation (3E) groups**.

	**Density of BrdU/NeuN cells**		**Percentage of BrdU/NeuN cells**	
	**aAOB**	**pAOB**	**aAOB**	**pAOB**
Ctr	5.9±1.7	4.4±1.9	40.11±10	36.3±13
3E	7±1.2	13.7±2.6^*^	47.6±9.6	63.6±6.7

* *Different form Ctr, P < 0.05*.

## Discussion

Our data reveal that those males that ejaculated three times pacing the sexual interaction show an increase in the density of new cells and neurons that survive in the granular cell layer (GrL) of the posterior accessory olfactory bulb (pAOB). No significant differences were found in the anterior accessory bulb (aAOB). Different kinds of socio-sexual stimulation can modulate adult neurogenesis differentially throughout the anterior and posterior regions of the AOB. Aggressive behavior in male mice increases neurogenesis in the aAOB, and female mice exposed to male urine show an increase in the number of new cells in the pAOB (Nunez-Parra et al., 2011). Thus, the pAOB plays a relevant role in detecting sexually relevant odors, and sexual stimulation increases neurogenesis in this structure.

We previously demonstrated that neither mating nor exposure to a sexually receptive female increased the density of new cells that arrive to the MOB. In the present study we confirmed these observations, because no increase in the density of new cells that survive in the MOB were found. In male rats, mating increased the early gene activity-regulated cytoskeleton-associated protein (Arc) immunoreactivity in the granular cells of the AOB. However, sexual activity did not increase the number of Arc-positive cells in the MOB (Matsuoka et al., 2002a,b). Similarly, in male rats exposure to estrous female bedding increased the number of c-Fos (Portillo and Paredes, 2004) and Arc-positive cells in the GrL and mitral cell layer of the AOB, but not in the MOB (Matsuoka et al., 2002b). Thus, in male rats, mating and estrous female odors are not very effective in activating the MOB.

In a previous study we found that those males that pace the sexual interaction and ejaculate once showed an increase in the density of cells that arrive at the AOB 15 days after the sexual interaction (Portillo et al., 2012). However, in the present study the stimulation provided by one ejaculation was not enough to increase the density of new cells that survive in the AOB when evaluated 45 days after mating. Studies in females have shown that those female rats that paced the sexual interaction before BrdU administration and then continued mating weekly for 3 additional weeks had a significantly higher density of new cells in the granular and mitral cell layers of the AOB and the MOB, suggesting that intense sexual stimulation increases the survival of new cells in different areas of the OB (Arzate et al., 2013). Further, studies need to address if repeated mating in males that control the sexual interaction increases the density of cells in different areas of the OB.

Although males from the non-pacing group ejaculate around three times (2.6 ± 0.4), this stimulation was not able to increase the survival of the new cells in the AOB. Similar results were found in our previous studies when we demonstrated that males not allowed to pace the sexual interaction did not show an increase in the density of cells that reach the AOB 15 days after sexual behavior (Portillo et al., 2012). One of the differences between pacing and no pacing is that only when the male and female rats pace the sexual interaction, sexual behavior induce a reward state, as evaluated by the conditional place preference (CPP) paradigm (Agmo and Berenfeld, 1990; Paredes and Alonso, 1997; Camacho et al., 2009; Arzate et al., 2011; Corona et al., 2011b). It has been demonstrated that rewarding experiences such as running and intracranial self-stimulation increase the proliferation, differentiation and survival of new cells in the dentate gyrus (Takahashi et al., 2009; Garrett et al., 2012). In rodents, the rewarding properties of paced mating depend on opioids since the administration of the opioid antagonist naloxone blocks the rewarding proprieties of CPP in male and female rats that pace the sexual interaction (Agmo and Gómez, 1993; García-Horsman et al., 2008). It has been demonstrated that opioids can regulate proliferation, gliogenesis, and neurogenesis (Narita et al., 2006; Chen et al., 2008). Opioid agonists decrease proliferation in the DG of the hippocampus and increase the survival of the new cells but do not modify the differentiation linage (Pettit et al., 2012). Preliminary data from our lab indicate that the increase in the density of new cells that reach the GrL of the AOB in female rats that paced the sexual interaction is inhibited by the i.p. administration of the opioid antagonist naloxone (unpublished data). Further, studies are needed to determine if naloxone can block the increase in the number of new cells that arrive and survive in the GrL of the AOB in those males that pace the sexual interaction, to determine if the neurogenesis induced by mating is opioid dependent.

It has been shown that in male Wistar rats, the first ejaculation increases the prolactin (PRL) levels in comparison to the precopulatory values. The increase in PRL levels reaches the highest point after the second ejaculation and start to decrease after the third one (Hernandez et al., 2006). PRL increases the SVZ and OB neurogenesis *in vivo* and *in vitro* (Bridges and Grattan, 2003; Shingo et al., 2003; Larsen et al., 2008; Walker et al., 2012). In male mice, the paternal-offspring interaction increases cell proliferation in the SVZ and DG and the number of new neurons that reach the OB and DG (Mak and Weiss, 2010). Disruption of PRL signaling by the administration of a PRL-neutralizing antibody and males with targeted disruption in the PRL receptor gene showed no increase in the neurogenesis induced by the paternal-offspring interaction (Mak and Weiss, 2010). Since males that ejaculate twice show the highest levels of PRL, it is possible that the elevated levels of this hormone are involved in the increase in the survival of new cells in the AOB of males that ejaculate three times. Female rats that mate in pacing conditions show an increase in PRL levels, whereas females that mate without pacing the sexual interaction do not (McClintock and Anisko, 1982; Erskine et al., 2004). Further, research is need to evaluate if in male rats that pace the sexual interaction PRL levels increase in comparison to those males that mate without pacing the sexual interaction.

Our data show that males that ejaculated three times pacing the sexual interaction showed an increase in the density of new cells that survive in the GrL of the pAOB and they also have more new neurons that the control animals. However, no differences were found in the percentage of new cells that differentiate into neurons. Glasper and Gould (2013) found similar results; they showed that retired breeders that mate consecutively for 14 days and stay sexually inactive for another 14 days did not increase the percentage of new cells that differentiate into neurons in the DG (Glasper and Gould, 2013). On the other hand, female rats that repeatedly mate showed an increase in the percentage of new neurons that reach the AOB (Arzate et al., 2013). Further, studies are needed to determine if in the female, but not in the male rat, sexual stimulation enhances the differentiation to the neuronal linage.

To summarize, the results of the present study show that male rats that ejaculate three times in one mating session have a higher density of cells and new neurons that survive in the GrL of the AOB 45 days after mating.

## Author contributions

NU, Performed the experiments and data analysis, help writing the paper; WP, Designed the study, help in writing the paper; RC, Help in the experiments; RP, Help in writing the paper and data analysis.

### Conflict of interest statement

The authors declare that the research was conducted in the absence of any commercial or financial relationships that could be construed as a potential conflict of interest.
